# Narrative Practice in Investigative Interviews of Individuals With Intellectual Disability

**DOI:** 10.1111/jar.70074

**Published:** 2025-06-13

**Authors:** Misun Yi

**Affiliations:** ^1^ Department of Policing and Forensic Psychology Dongyang University Yoonguju‐Si Republic of Korea

**Keywords:** intellectual disability, investigative interview, narrative practise, National Institute of Child Health and Human Development protocol

## Abstract

**Background:**

Individuals with intellectual disabilities are amongst the groups most vulnerable to sexual crimes. However, their limited cognitive abilities can make it challenging to obtain detailed statements from victims during investigative interviews. This study examined whether practising answering open‐ended questions early in the interview increases the accuracy and abundance of incident‐related information provided by individuals with intellectual disability.

**Method:**

Forty‐eight adults with intellectual disabilities and 32 children without disabilities aged 5–7 were interviewed about a photography event.

**Results:**

When practising with open‐ended questions, adults with intellectual disability and children without disabilities gave greater detail than those whose practise narrative involved specific questions. Furthermore, both groups provided more information in response to open‐ended questions. No effects were observed for inaccurate details.

**Conclusion:**

Narrative practices could afford individuals with intellectual disability the opportunity to familiarise themselves with open‐ended prompts, enabling them to provide more information during the substantive phase of the interview.

**Summary**

- Narrative practice enhances information recall: Narrative practices before the substantive interview phase improved the quality and quantity of information recalled by both individuals with intellectual disabilities and children without disabilities. This suggests that providing individuals with intellectual disabilities the opportunity to practise answering open‐ended questions can significantly benefit the interview process and the outcomes of investigative interviews.
- Open‐ended questioning increases information accuracy: Individuals with intellectual disability and children without disabilities provided more accurate and abundant information when asked open‐ended questions, compared to specific questions. This highlights the importance of using open‐ended prompts in investigative interviews to elicit more detailed and reliable information.
- Need for tailored interview approaches: This study underscores the need for tailored interview approaches for individuals with intellectual disability, considering factors like severity of disability and communication skills. Further research is needed to explore effective interview strategies for individuals with severe disabilities, ensuring their needs are adequately addressed in investigative interviews.

## 1 Introduction

Individuals with intellectual disability are one of the groups most vulnerable to sexual assault (Fisher et al. 2016; Tomsa et al. 2021). The lifetime prevalence of sexual assault amongst individuals with intellectual disability is approximately 7%–34% (Tomsa et al. 2021), which is about one and a half to two times as high as that of their counterparts without intellectual disability (Jones 2007). Further, the sexual abuse of children with intellectual disability tends to cause severe (Hershkowitz et al. 2007; McCarthy et al. 2022) and chronic damage that persists into adulthood (Sobsey and Doe 1991). Sexual assault incidents involving individuals with intellectual disability are seldom disclosed to others (Chave‐Cox 2014; Olley and Cox 2021).

Unfortunately, individuals with intellectual disabilities face challenges in understanding legal processes and effectively participating in their defence due to difficulties with comprehension, communication, language impairments and maladaptive behaviours (Hauser and Kohn 2024). Intellectual disability is associated with deficits in memory capacity (Danielsson et al. 2012; Kail 1990; Swanson and Trahan 1990) and poor verbal and comprehension skills (Tuffrey‐Wijne and McEnhill 2008). These symptoms can hamper effective communication with investigative interviewers and prevent investigators from obtaining all the information they need. Moreover, high suggestibility (Ceci and Bruck 1993; Gudjonsson and Henry 2003; Kebbell and Hatton 1999) and the tendency to acquiesce (Beail 2002; Heal and Sigelman 1995; Milne and Bull 2006; Perry and Felce 2004) can cause victims with intellectual disability to be perceived as unreliable sources of information (Brown and Lewis 2013; Henry et al. 2011).

For this reason, their cases are less likely to progress through the legal system (Olley and Cox 2021). In such cases, the rate of prosecution is low (Clare and Murphy 2001; Sobsey and Doe 1991), and court sentences are less severe than for cases involving female victims without disabilities (Bryen 2002; Sobsey and Doe 1991; Jantjies 2023; Yi 2020; Yi and Jung 2020). Given these challenges, it is critical to explore ways to improve the accuracy and informativeness of investigative interviews with individuals with intellectual disabilities. The present study aims to examine the effects of practising responses to open‐ended questions prior to the substantive interview on the quality and quantity of information provided by adults with intellectual disabilities.

## 2 Investigative Interviews With Individuals With Intellectual Disabilities

It is crucial for investigative interviewers to understand what happened during a criminal incident to inform the investigation and trial process. The type of questions posed during interviews significantly influences the accuracy and informativeness of the responses provided. Open‐ended questions, which are input‐free prompts encouraging detailed, free‐recall responses, consistently elicit longer and more comprehensive answers compared to specific questions (Davies et al. 2000; Hershkowitz 2001; Lamb et al. 2002; Orbach et al. 2000; Sternberg et al. 1997).

Types of specific questions, which direct respondents' attention to particular details or aspects of an event and often require focused responses rather than open‐ended recall, include directive, option‐posing and suggestive formats (Lamb et al. 2002; Hershkowitz 2001; Milne and Bull 2002). Directive questions encourage interviewees to elaborate on specific aspects of an incident already mentioned, relying on recall memory. Option‐posing questions, in contrast, limit responses to “yes” or “no” answers or a choice amongst given options, which can reduce accuracy if interviewees guess or respond without fully understanding the question (Cassel et al. 1996; Lamb and Fauchier 2001; Milne and Bull 2002). Suggestive questions present an even greater risk as they imply a particular response or introduce information not provided by the respondent, potentially compromising accuracy or generating false memories (Cassel et al. 1996; Lamb and Fauchier 2001).

Research has shown that suggestive questions tend to elicit high levels of misinformation from individuals with mild intellectual disabilities, particularly those that imply certain information to be true (Bowles and Sharman 2014). In light of these findings, the use of open‐ended questions has been consistently emphasised to enhance the accuracy and depth of the information provided by individuals with intellectual disabilities(Agnew and Powell 2004; Brown et al. 2017; Cederborg and Lamb 2008; Gordon et al. 1994).

However, investigative interviewers often lack adequate training in techniques specifically tailored to individuals with intellectual disabilities (Cederborg and Lamb 2008). As a result, they may avoid using open‐ended questions, instead favouring focused questions that elicit brief or yes/no responses (Åker and Johnson 2020; Brown et al. 2017). Furthermore, interviewers frequently interrupt individuals with intellectual disabilities to seek clarification of their narrative responses (Agnew et al. 2006; Cederborg and Lamb 2008; Cederborg et al. 2008), and they tend to adjust their questioning strategies based on the responsiveness of the interviewee (Gilstrap and Ceci 2005). Therefore, the effectiveness of investigative interviews with individuals with intellectual disabilities relies on both the interviewer's skill in employing appropriate techniques and the interviewee's ability to engage with open‐ended prompts.

Narrative practices aims to afford interviewees the opportunity to practise answering open‐ended prompts. It allows interviewees to prepare for investigative interviews by familiarising them with the strategies used before discussing the target events. Through practising the description of episodic events by engaging with questions similar to those that will be used during the actual interview, interviewees not only learn the level of detail expected in their answers but are also encouraged to expand on the event‐specific details they provide (Brubacher et al. 2011; Roberts et al. 2011; Lamb et al. 2018; Sternberg et al. 1997). In addition to preparing interviewees for the interview structure, narrative practices also plays a critical role in building rapport, fostering a sense of being heard, and helping interviewers assess the interviewees' expressive language abilities. Moreover, performance during narrative practice has been shown to reliably predict how interviewees will respond during the substantive phase, making it a valuable tool not only for engagement but also for tailoring the interview approach accordingly (Bearman et al. 2022).

Previous studies have consistently demonstrated that practise in answering open‐ended questions, as opposed to specific or option‐posing questions, during the narrative phase results in more abundant yet equally accurate information about the event in question from children (Brown and Lewis 2013; Foster et al. 2023; Price et al. 2013; Brubacher et al. 2011; Roberts et al. 2004, 2011; Sternberg et al. 1997; Whiting and Price 2017; Yi and Lamb 2018). Expanding on these findings, Bearman et al. (2022) specifically examined adults with limited expressive language and found that they were able to respond meaningfully to open‐ended questions about personal experiences. Despite using brief utterances, many participants demonstrated the ability to provide coherent and informative narratives. Importantly, narrative performance during the practice phase significantly predicted performance in the substantive phase, suggesting that narrative practice can serve as an effective tool for assessing communication abilities and preparing interviewees for investigative questioning.

## 3 The Purpose of the Study

The purpose of this study is twofold. First, it aimed to show that practise recalling episodic events in response to open‐ended prompts rather than specific prompts is more likely to elicit accurate and abundant information from individuals with intellectual disability when substantive issues are discussed. Although the effects of narrative practice on children's informativeness have been repeatedly confirmed, little is known about whether narrative practice could enhance accounts elicited by adult witnesses with intellectual disabilities. Open‐ended questions do not specify which details should be retrieved (Lamb et al. 1996). Interviewees may find it difficult to answer such prompts if they do not understand the intentions of the interviewer (e.g., the kind and extent of information they are being asked to provide). Individuals with intellectual disabilities usually have less opportunity to discuss their past experiences with others compared to their counterparts without intellectual disability (Hatton 1998). As a result, they may find it more difficult to recall details about episodic events in response to open‐ended prompts. In addition, adults with intellectual disabilities face unique challenges in interviews due to societal expectations surrounding adult behaviour. Research suggests that they may feel compelled to align their responses with perceived expectations from authority figures, which can make them more vulnerable to suggestibility (Åker and Johnson 2020; Clare and Gudjonsson 1995; Griego et al. 2019). Therefore, it is expected that providing individuals with intellectual disabilities the opportunity to practise answering open‐ended questions will significantly increase their ability to take responsibility when discussing targeted events.

Second, this study aimed to examine the effect of the type of question on the information elicited from mock witnesses. Open‐ended questions may elicit more information from children with or without intellectual disability than focused questions (Brown et al. 2017; Cederborg and Lamb 2008; Kebbell and Hatton 1999). However, when working with very young children (Andrews et al. 2016; Hershkowitz et al. 2012; Stolzenberg et al. 2017) and individuals with moderate intellectual disability (Hershkowitz 2018), focused questions might be more effective than open‐ended questions. The effectiveness of open‐ended questions in eliciting information may also differ based on the intelligence levels of interviewees. Few studies have focused on the effects of the type of question on the accuracy and abundance of information elicited from adults with intellectual disability (Bearman et al. 2019; Bowles and Sharman 2014; Cederborg et al. 2012; Hershkowitz 2018). Therefore, this study investigated the effect of the type of question asked on the quality and quantity of information provided by adults with intellectual disability in South Korea.

## 4 Materials and Methods

The study involved 48 adults with intellectual disabilities and 32 children without disabilities aged 5–7 to assess whether narrative practices, which was designed to elicit more accurate and abundant information from child victims, would also be effective for individuals with intellectual disabilities. Data collection spanned two periods: November 2019 to January 2020 and September 2023. Initially, nine daycare centres for individuals with intellectual disabilities agreed to participate in the study, but three centres cancelled due to the COVID‐19 pandemic lockdowns that coincided with the scheduled interview session. During the initial data collection period, 49 adults with intellectual disabilities from six daycare centres for people with intellectual disabilities agreed to participate in the experiment. However, six individuals either refused to participate or did not appear on the appointed day. Furthermore, two participants declined to continue their involvement in the experiment midway through the proceedings. During the subsequent data collection session, 20 individuals with intellectual disabilities from two daycare centres agreed to participate, but only 13 attended the interview session. Consequently, data were ultimately collected from a total of 56 participants. Some participants' data were excluded from the analysis for the following reasons: Two participants—one with a hearing impairment and another with a visual impairment—were excluded due to their inability to acquire the same amount of information as their counterparts. Additionally, six individuals were excluded from analysis because they were not cooperative: They said little or nothing during the narrative practise phase, irrespective of the type of questions they were asked. Therefore, the final sample comprised 48 individuals.

Notably, amongst the excluded participants, five were assigned to the narrative practice group with open‐ended questions, and one was assigned to the narrative practice group with specific questions. It cannot be excluded that the nature of the questions during the narrative practices phase may have influenced non‐cooperative responses. These issues will be thoroughly addressed as limitations in the discussion.

The participants were between 20 and 67 years old, with a mean age of 38.1 years (SD = 12.17). Twenty‐two participants (45.8%) were men, and 26 (54.2%) were women. They were all diagnosed with intellectual disabilities and registered in the national system; however, their intelligence quotient scores were not verified. Eight individuals (16.7%) were recruited from Centre A, 10 (20.8%) from Centre B, 3 (6.3%) from Centre C, 2 (4.2%) from Centre D, 3 (6.3%) from Centre E, 8 (16.7%) from Centre F, 8 (16.7%) from Centre G, and 6 (12.5%) from Centre H. Participants from each centre were evenly assigned across conditions, so participants from a particular centre were not assigned significantly more often to either narrative practise condition, Χ2 (7, *n* = 48) = 0.830, *p* = 0.997.

The comparison group was comprised of 32 children without disabilities who were between 5 and 7 years old. The parents of 40 children without disabilities provided consent for participation. However, seven were absent on the day of the experiment, and one child was uncooperative. Consequently, the final sample comprised 32 children. Notably, 18 (56.3%) were boys, and 14 (43.7%) were girls. Their chronological ages were 5 (*n* = 5, 16.6%), 6 (*n* = 17, 53.1%) and 7 years (*n* = 10, 31.3%).

Seven participants (21.8%) were recruited from nursery A, and 25 (78.1%) were recruited from nursery B. The child participants from each nursery were evenly assigned to the narrative practise conditions, *Χ* ^*2*^(1, *n* = 32) = 0.001, *p* = 0.652.

### 4.1 Procedure

To recruit participants, researchers searched the websites of daycare centres for individuals with intellectual disability in South Korea. Then, the researchers called these centres, informed them about the purpose of the study and its procedures, and asked if they could conduct the study with those who came to their respective centres. Those that agreed were sent a written proposal and consent forms so that the legal guardians of those who came to their facility could provide consent. Out of over 50 centres contacted, only 8 ultimately agreed to participate. To recruit children without disabilities, I contacted a few nurseries and requested their participation using the same method that was used to recruit individuals with intellectual disability. Two nurseries agreed to take part in the study.

The experiment comprised three sequential phases: (1) a photography event, (2) a structured interview session and (3) a debriefing session. All procedural components transpired within the confines of the originating centre of participant recruitment. One room was set up as a photo studio. It contained only a large fabric cartoon poster and a video camera placed opposite the poster, which recorded the entire photography event. The poster, which depicted animals and trees, was hung on a wall to serve as the background for the photographs taken during the experiment. Two other rooms served as the interview rooms, where the participants were interviewed about what had happened during the photography event. Each interview room contained one table and two chairs. The interviews were audio recorded. Immediately after the interview session, the participants were debriefed in the same interview room.

#### 4.1.1 Photography Event Paradigm

The photography event paradigm used in this study was adapted from a procedure used in past studies (Brown and Lewis 2013; Roberts et al. 1999; Yi and Lamb 2018). In the experiment, a female confederate served as the photographer, and she took photos of the participants following tightly scripted procedures. For each photography event, the photographer helped the participant put on a cowboy costume, which comprised a hat, a vest, a scarf and a gun. At the end, the photographer requested the participant to take a photo of her (i.e., the photographer). Further, she warned them, “You cannot tell anyone that you took a picture of me. I am the only person who can touch the camera. I will be in trouble if someone finds out that you touched the camera.” (See Yi and Lamb 2018, for more details). The event lasted for approximately 5 min, and all the procedures were video recorded. The participants were instructed to not discuss the event but to proceed with their daily schedules until they were called to be interviewed.

#### 4.1.2 Interview Session

The interview session was conducted approximately 2–3 h after the photography event ended. Two female interviewers conducted the interviews in separate rooms. The participants were randomly assigned to one of the two interviewers. Further, the participants were randomly assigned to one of two narrative practice conditions: (1) practice with open‐ended questions, or (2) practice with specific questions. In the beginning, each interviewer introduced herself and explained ground rules, which included instructions to say “I don't know,” “I don't remember,” and to correct the interviewer when needed. Those assigned to the open‐ended question group were asked 10 open‐ended questions about what they did over the last weekend, whereas those assigned to the specific question group were asked 10 specific questions on similar topics. Then, all the participants were asked 7 open‐ended, 7 specific and 7 misleading questions about what had happened during the photography event. When a participant did not answer a question or said, “I don't know” or “I don't remember,” the interviewer encouraged him or her to answer the question by saying, “You can take your time and answer the question when you are ready” or “You can tell me whatever comes to mind.” When these prompts failed to elicit a response, the interviewer gave them another chance to answer the question by providing the same prompts. If that failed, the interviewer proceeded to the next question. Finally, the interviewees were thanked, and the interview was terminated. The interview lasted for approximately 30 min. All the interviews were audio‐recoded and subsequently transcribed for analysis. All questions posed during the narrative practice and substantive interview phases presented in Appendix A.

#### 4.1.3 Debriefing

Perpetrators often ask their victims to keep their acts of sexual abuse a secret. In this regard, the fact that the photographer had asked the participants to keep what had happened between them a secret enhances the ecological validity of the experiment. However, this may have evoked negative emotions in the participants. The guardians were informed about this possibility through the consent form. Moreover, immediately after the interview, the participants were informed that they could touch the camera and that nothing would happen to them if they were to touch it. Finally, each participant received a pack of snacks and a gift card worth 10,000 Korean won as compensation. After all the experimental procedures were completed, a thank you card and a framed photo of the participant wearing the cowboy costume taken during the experiment were mailed to each participant. All experimental procedures were reviewed and approved by the Institutional Review Board in South Korea (1041495‐201908‐HR‐01‐01).

### 4.2 Analysis

The informativeness of participant responses was assessed in terms of the number of accurate and inaccurate details provided. The number of reported details was recorded in a coding system developed by Lamb et al. (2007) and adapted for the Korean context by Yi and Lamb (2018). Modifications were mostly related to the use of articles and “be” verbs. The Korean language does not use articles except for emphasis. Nor does it use “be” verbs; prepositions are transformed into verbs by adding a suffix (i.e., “da,” “eda”) to the preposition. For example, Koreans just say “Hat red‐eda” instead of “The hat is red,” and in both Korean and English, details are counted as two.

A detail was defined as any piece of information that pertained to the event, and it was counted based on the number of words. For example, one participant said, “I wore a brown hat.” This description was related to the event, and it included four details (I/wore/brown/a hat). Meaningless interjections (e.g., “um”), gibberish, and information unrelated to the event were not counted. Each detail was counted only once (i.e., when first mentioned). Therefore, pronouns were largely not counted. However, a piece of information was counted again, when it was reported in response to a different type of question (open‐ended, specific, or misleading).

Each detail was coded as accurate or inaccurate. Accurate details were correct recollections and synonymous descriptions of correct details. For example, if the colour of the hat was brown, “brown,” “mocha,” and “chocolate colour” were considered to be accurate. When a participant described emotions and thoughts experienced during the event, this detail also was coded as accurate because they are subjective. Incorrect details, source‐monitoring errors, and errors of commission were coded as inaccurate. For example, if the participant had worn a brown hat but said, “I wore a red cap,” it was coded as follows: 2 accurate details (I/wore) and 2 inaccurate details (red/a cap) (for more details, see Lamb et al. 2007; Yi and Lamb 2018). The raters watched the video clips when they could not determine the accuracy of the information provided by a participant. Details communicated during the narrative practise phase were counted but not assessed for accuracy.

There was a high level of inter‐rater reliability between the number of accurate and inaccurate details reported by two independent raters. Each of them coded the same 12 cases separately for reliability analysis. For accurate and inaccurate details, the ICC coefficients were 0.992 (95% confidence interval = 0.989–0.994), *F* (219, 219) = 235.44, *p* < 0.001, and 0.998 (95% confidence interval = 0.997–0.998), *F* (219, 219) = 907.81, *p* < 0.001, respectively.

## 5 Results

### 5.1 Manipulation Check

Repeated‐measures analysis of variance confirms the informativeness during the narrative practise phase as a function of disability and the type of question during the narrative practises. The number of details elicited from the first question was used as a baseline to compare the mean number of details from the last nine narrative practise questions (questions 2 to 10). Results showed that before the narrative practise phase began, there were no differences in details based on the conditions for the narrative practice, *t* (79) = 0.177, *p* = 0.860. However, the mean number of details increased when the participants practised with open‐ended questions, *F* (1, 77) = 16.031, *p* < 0.001, *η*_*p*_^*2*^ = 0.172. There were no significant interaction effects of narrative practice and disability group, *F* (1, 77) = 1.729, *p* = 0.192, *η*_*p*_^*2*^ = 0.022, nor three‐way interactions on the number of details, *F* (1, 77) = 0.046, *p* = 0.831, *η*_*p*_^*2*^ = 0.001 (see Tables 1 and 2). The results suggest that manipulated variables indeed affect the dependent variable as intended.

**TABLE 1:** Mean number of details as a function of disability and narrative practise during the narrative practises.

Disability	Narrative practice	*n*	Details	
			1st question	Mean details
ID group	With open‐ended questions	21	4.33 (3.25)	8.68 (6.58)
	With specific questions	27	4.92 (3.71)	3.62 (2.01)
	Total	48	4.66 (3.49)	5.84 (5.21)
TD children	With open‐ended questions	14	8.50 (5.66)	11.21 (8.92)
	With specific questions	18	6.89 (11.13)	3.31 (2.04)
	Total	32	7.58 (9.13)	6.66 (7.10)
Total	With open‐ended questions	35	6.00 (4.77)	9.69 (7.58)
	With specific questions	45	5.74 (6.74)	3.49 (2.01)
	Total	80	5.85 (6.53)	6.17 (6.02)

Abbreviations: ID, intellectual disability; TD, typically developing.

**TABLE 2:** Results of repeated‐measures analysis of variance for the effects of question type and disability group on the number of details during the narrative practice phase.

		SS	d*f*	MS	*F*	*p*	*η*_p_^2^
Question type		11.242	1	11.242	0.527	0.470	0.007
Question type × disability group		36.870	1	36.870	1.729	0.192	0.022
Question type × details		341.804	1	341.804	16.031	0.000	0.172
Question type × disability group × details		0.973	1	0.973	0.046	0.831	0.001
Error		1641.732	77	21.321			

*Note:* Mauchly's test results showed that the assumption of sphericity had been established, χ^2^(0) = 0.000, n.s., *ɛ* = 1.000.

### 5.2 The Quality and Quantity of Details as a Function of Narrative Practice and Disability

Multivariate analysis of variance was conducted to determine whether the narrative practice affected the quality and quantity of details during the substantive interview session. Participants who practised answering open‐ended questions before the substantive interview (*M* = 31.94, SD = 22.93) provided significantly more accurate details about the event in question than those who practised answering specific questions (*M* = 21.41, SD = 18.30), *F* (1, 76) = 7.492, *p* = 0.008, *η*_p_^*2*^ = 0.090. Children without disabilities (*M* = 33.02, SD = 16.92) also provided more accurate details than participants with intellectual disability (*M* = 21.35, SD = 17.85), *F* (1, 68) = 9.124, *p* = 0.003, *η*_p_^2^ = 0.107, but the interaction effect was not significant, *F* (1, 76) = 0.017, *p* = 0.896, *η*_p_^2^ = 0.000. Hence, practising answering open‐ended questions during the narrative practice phase increased the amount of accurate details that both adults with intellectual disability and children without disabilities provided. Regarding the amount of inaccurate details, there was no significant difference between the narrative practice groups (open‐ended questions: *M* = 7.72, SD = 5.12, specific questions: *M* = 7.54, SD = 7.34; *F* [1, 76] = 0.021, *p* = 0.885, *η*_p_^2^ = 0.000) or between individuals with intellectual disability (*M* = 8.31, SD = 7.50) and children without disabilities (*M* = 6.59, SD = 7.47), *F* (1, 68) = 0.600, *p* = 0.441, *η*_p_^2^ = 0.009. The interaction effect was also not significant, *F* (1, 76) = 0.013, *p* = 0.911, *η*_p_^2^ = 0.000 (see Tables 3 and 4).

**TABLE 3:** Mean number of accurate and inaccurate details as a function of disability and narrative practise.

Detail type	Disability	Narrative practise	*n*	*M*	(SD)
Accurate	ID	With open‐ended questions	21	27.05	(23.03)
		With specific questions	27	16.93	(11.02)
		Total	48	21.35	(17.85)
	TD	With open‐ended questions	14	39.28	(21.51)
		With specific questions	18	28.15	(10.51)
		Total	32	33.02	(16.92)
	Total	With open‐ended questions	35	31.94	(22.93)
		With specific questions	45	21.41	(12.01)
		Total	80	26.02	(18.30)
Inaccurate	ID	With open‐ended questions	21	8.33	(4.20)
		With specific questions	27	8.28	(6.57)
		Total	48	8.31	(7.50)
	TD	With open‐ended questions	14	6.81	(6.31)
		With specific questions	18	6.43	(8.45)
		Total	32	6.59	(7.47)
	Total	With open‐ended questions	35	7.72	(5.12)
		With specific questions	45	7.54	(7.34)
		Total	80	7.62	(6.42)

Abbreviations: ID, intellectual disability; TD, typically developing.

**TABLE 4:** Analysis of variance results for the effect of disability and narrative practice on the total number of accurate and inaccurate details.

Factor (s)	Detail type	SS	d*f*	MS	*F*	*p*	*η*_p_^2^
Disability group	Accurate	4800.366	1	2600.576	9.124	0.003	0.107
	Inaccurate	57.449	1	54.039	1.280	0.261	0.017
Narrative practise group	Accurate	2135.493	1	2135.493	7.492	0.008	0.090
	Inaccurate	0.886	1	0.886	0.021	0.885	0.000
Disability group × Narrative practise group	Accurate	4.876	1	4.876	0.017	0.896	0.000
	Inaccurate	0.528	1	0.528	0.013	0.911	0.000
Error	Accurate	21661.489	76	285.020			
	Inaccurate	3208.716	76	42.220			
Total	Accurate	80628.556	80				
	Inaccurate	7912.333	80				

*Note:* Mauchly's test results showed that the assumption of sphericity had been established, χ^2^(0) = 0.000, n.s., *ɛ* = 1.000.

Subsequent independent T‐tests were conducted to validate the effectiveness of the question types used during the narrative practice phase within the intellectual disability group, with the typically developing group eliminated as a controlled factor. The findings revealed that those participants with intellectual disability who practised answering open‐ended questions provided significantly more details than those who practised answering specific questions, *t* (46) = 2.011, *p* = 0.050, without the amount of inaccurate details increasing, *t* (46) = 1.137, *p* = 0.261.

### 5.3 The Quality and Quantity of Details as a Function of the Question Type

Repeated‐measures analysis of variance was conducted, with the total number of accurate and inaccurate details elicited via open‐ended, directive, and misleading questions as the dependent variables (see Tables 5, 6, 7). Amongst the participants with intellectual disability and the children without disabilities, open‐ended questions elicited the most accurate details, whereas misleading questions elicited the fewest, *F* (3, 249) = 69.375, *p* < 0.001, *η*_p_^2^ = 0.455. Further, children without disabilities provided more details than those in the intellectual disability group in response to open‐ended questions, *F* (1, 79) = 6.192, *p* = 0.015, and directive questions, *F* (1, 79) = 6.823, *p* = 0.011, but no differences were found in the amount of details given in response to suggestive questions between the typically developing and the intellectual disability groups, *F* (1, 46) = 3.048, *p* = 0.085.

**TABLE 5:** Mean number of accurate details as a function of question type.

Participant group	Narrative practice group	Details	*M*	(SD)
ID	With open‐ended questions (*n* = 21)	Open‐ended	42.24	(46.33)
		Specific	21.57	(18.59)
		Misleading	12.33	(11.59)
	With specific questions (*n* = 27)	Open‐ended	25.04	(21.66)
		Specific	15.67	(9.54)
		Misleading	18.65	(11.68)
	Total (*n* = 48)	Open‐ended	34.75	(36.01)
		Specific	18.25	(14.36)
		Misleading	11.06	(9.54)
TD	With open‐ended questions (*n* = 14)	Open‐ended	62.64	(34.37)
		Specific	39.36	(34.59)
		Misleading	15.86	(8.81)
	With specific questions (*n* = 18)	Open‐ended	46.32	(19.54)
		Specific	22.89	(13.31)
		Misleading	13.74	(8.01)
	Total (*n* = 32)	Open‐ended	53.24	(27.60)
		Specific	29.88	(25.57)
		Misleading	14.64	(8.29)

Abbreviations: ID, intellectual disability; TD, typically developing.

**TABLE 6:** Mean number of inaccurate details as a function of question type.

Participant group	Narrative practice	Question type	*M*	(SD)
ID	With open‐ended questions (*n* = 21)	Open‐ended	3.52	(4.99)
		Specific	11.19	(9.82)
		Misleading	10.28	(5.27)
	With specific questions (*n* = 27)	Open‐ended	5.96	(8.71)
		Specific	8.26	(5.71)
		Misleading	10.63	(11.23)
	Total (*n* = 48)	Open‐ended	4.89	(7.38)
		Specific	9.54	(7.82)
		Misleading	10.48	(9.03)
TD	With open‐ended questions (*n* = 14)	Open‐ended	4.57	(6.24)
		Specific	8.21	(6.31)
		Misleading	7.64	(10.16)
	With specific questions (*n* = 18)	Open‐ended	3.33	(3.99)
		Specific	7.39	(8.94)
		Misleading	8.56	(14.54)
	Total (*n* = 32)	Open‐ended	3.88	(5.04)
		Specific	7.75	(7.79)
		Misleading	8.16	(12.63)

Abbreviations: ID, intellectual disability; TD, typically developing.

**TABLE 7:** Repeated‐measures analysis of variance results for the effect of question type and disability on the number of accurate and inaccurate details.

Detail type	Factor (s)	SS	d*f*	MS	*F*	*p*	*η*_p_^2^
Accurate	Question type	41618.891	3	13872.964	69.375	0.000	0.455
	Question type × disability group	2528.595	3	842.865	4.215	0.006	0.048
	Question type × narrative practise group	2059.171	3	686.390	3.432	0.018	0.040
	Question type × disability group × narrative practise group	574.296	3	191.432	0.957	0.414	0.011
	Error	49792.856	249	199.971			
Inaccurate	Question type	1110.591	2	555.296	11.954	0.000	0.136
	Question type × disability group	24.749	2	12.374	0.266	0.766	0.003
	Question type × narrative practise group	78.3	2	39.150	0.843	0.433	0.011
	Question type × disability group × narrative practise group	84.790	2	42.395	0.913	0.404	0.012
	Error	7060.780	152	46.452			

*Note:* Mauchly's test results showed that the assumption of sphericity had been established, χ^2^(2) = 2.679, n.s., *ɛ* = 0.965.

Narrative practices significantly affected the amount of details elicited via each question type utilised during the substantive interview phase, *F* (3, 249) = 3.432, *p* = 0.018, *η*_p_^2^ = 0.040. Specifically, when narrative practice involved open‐ended questions, more details were elicited in response to open‐ended questions, *F* (1, 79) = 7.128, *p* = 0.009, and directive questions, *F* (1, 79) = 5.057, *p* = 0.027, but when narrative practise involved open‐ended questions, the amount of details given in response to misleading questions did not increase, *F* (1, 79) = 1.101, *p* = 0.297.

The highest amount of inaccurate details was elicited via misleading questions (participants with intellectual disability: *M* = 10.48, SD = 9.03; children without disabilities: *M* = 8.16, SD = 12.63), followed by directive questions (participants with intellectual disability: *M* = 9.45, SD = 7.82; children without disabilities: *M* = 7.75, SD = 7.79) and open‐ended questions (participants with intellectual disability: *M* = 4.89, SD = 7.38; children without disabilities: *M* = 3.88, SD = 5.04), *F* (2, 152) = 11.954, *p* < 0.001, *η*_p_^2^ = 0.136. The interaction effects of disability, *F* (2,152) = 0.266, *p* = 0.776, *η*_p_^2^ = 0.003, and narrative practise, *F* (2, 152) = 0.843, *p* = 0.404, *η*_p_^2^ = 0.012, were not significant.

## 6 Discussion

This study proposed to examine whether practising answering open‐ended rather than specific questions during the early phases of an interview increases the accuracy and abundance of incident‐related information that individuals with intellectual disability provide. The results suggest that before the narrative practice phase, adults with intellectual disability and children without disabilities provide similar amounts of details, regardless of the practice conditions, but the amount of details significantly increases when both adults with intellectual disability and children without disabilities are asked open‐ended questions. Meanwhile, the amount of details given during the practise phase is similar to or even less than the amount of details given before the practice phase when participants are repeatedly asked specific questions about their experience. Furthermore, narrative practices increases the quality and quantity of information provided by adults with intellectual disability and children without disabilities when they are asked to recall events. In terms of accurate and inaccurate details, children without disabilities provided more accurate statements than individuals with intellectual disabilities, although no significant differences were found in inaccurate details. Furthermore, it was found that the group who practised responding to open‐ended questions during the narrative practice session provided more accurate details than the group who practised answering specific questions—without an accompanying increase in inaccurate details. These results align with those of previous studies (Brown and Lewis 2013; Brubacher et al. 2011; Price et al. 2013; Roberts et al. 2004; Sternberg et al. 1997; Yi and Lamb 2018), which showed that allowing individuals with intellectual disability to practise answering open‐ended questions early in an interview increases the number of accurate details they provide without increasing the number of inaccurate details they provide.

Narrative practices has several benefits for the process and outcomes of investigative interviews with individuals with intellectual disability. First, individuals with intellectual disability may find it difficult to answer the types of questions that are often posed during investigative interviews. Specifically, open‐ended prompts do not inform the interviewee of the nature and extent of the information that they are required to provide, making it difficult for them to understand the purpose of such questions. Narrative practices affords individuals with intellectual disability the opportunity to familiarise themselves with open‐ended prompts, enabling them to provide more information during the substantive phase of the interview. In addition, narrative practise helps interviewers establish rapport with witnesses with intellectual disability. A previous study found that interviewees who were interviewed following the National Institute of Child Health and Human Development (NICHD) protocol that incorporates narrative practices were more satisfied with their interviewer and the interview process than those who were interviewed following the general NICHD protocol (Yi et al. 2017). This is because speaking about their experiences may help individuals with intellectual disability feel safe, perceive the interviewer as trustworthy, and be more responsive. Finally, narrative practise may benefit investigative interviewers by allowing them to practise posing open‐ended questions before they explore the details of the incident under investigation (Lamb et al. 2018).

Both individuals with intellectual disabilities and children without disabilities provide more abundant information in response to open‐ended questions than in response to directive or misleading questions. The amount of inaccurate details provided also remains the same. Consistent with past observations, these findings underscore the usefulness of open‐ended questions in eliciting information not only from children (Agnew and Powell 2004; Agnew et al. 2006; Brown et al. 2017; Cederborg and Lamb 2008) but also from individuals with intellectual disability (Hershkowitz 2018).

However, using open‐ended prompts in real‐world practice is a complex undertaking. Unlike what happens in experiments, sexual abuse cases are often disclosed after a considerable amount of time has passed. After the event is disclosed, there may also be a delay in conducting the first investigative interview. Moreover, because such incidents tend to be traumatic and difficult to understand, victims with intellectual disability may find it challenging to disclose details about the event in response to recall prompts, whilst the usefulness of open‐ended prompts is likely to be limited or even totally ineffective when interviewing individuals with severe disabilities and communication problems.

Some limitations of this study should be addressed. First, this study could not use the severity of participants' disability as a variable. Previous studies have shown that the accuracy and amount of information provided in response to different types of questions differ according to the severity of the participant's disability (Brown et al. 2017; Cederborg and Lamb 2008; Kebbell and Hatton 1999; Hershkowitz 2018). That is, individuals with mild intellectual disabilities, compared with those with moderate intellectual disabilities, provide more accurate and abundant information when asked open‐ended questions. In addition, the abilities of participants, including verbal skills and social competencies, may have influenced the results. Although all participants possessed a nationally recognised disability certificate, specific information regarding individual abilities could not be verified. Whilst participants were randomly assigned to conditions in order to minimise the potential impact of personal characteristics on the research outcomes, this limitation warrants further consideration. Future studies should thus compare the effects of using different types of narrative practices and interview questions in relation to the severity of the disability and participants' abilities. Second, this study focused only on individuals with intellectual disabilities who had been attending a daycare centre regularly. Therefore, they may have had better communication and social skills than those without regular social interactions.

Second, a significant limitation of this study is the comparison between adults with intellectual disabilities and children without disabilities aged 5–7. Even when considering estimated mental age (Kaufman and Kaufman 2001), this comparison may not be appropriate as it overlooks the developmental differences between these groups. A more suitable approach would have been to compare adults with intellectual disabilities to typically developing adults or children of an older age range. This limitation should be carefully considered when interpreting the results, as it may affect the validity of the findings and their applicability to real‐world contexts.

Third, the current study is limited by the potential difference in the length of time between the two narrative practice conditions, with the specific question condition possibly being shorter. This could influence both rapport building and participant fatigue, although these factors may counterbalance each other. Future studies should consider the optimal duration of narrative practices and its potential effects on interview outcomes.

Further, the effectiveness of the narrative practice phase in eliciting information from those with severe disabilities remains unclear. The participants may also have found it easier to remember and respond to questions about the photography event because (a) it was a one‐time occurrence, (b) their interaction with the photographer was relatively brief and (c) the interview was conducted within a few hours following the event. In criminal cases, by contrast, the interval between the incident and interview is usually much longer, and victims may find it difficult to recall specific details about the incident.

Finally, more participants assigned to the narrative practice with open‐ended questions were excluded compared to those who were asked specific questions. Hence, the results suggest that the narrative practice phase may not be as effective for some individuals with intellectual disabilities since it may be difficult for them to practise answering open‐ended questions, and this may eventually cause them to give non‐cooperative responses. Strategies to enhance the responsiveness of individuals with intellectual disabilities who have difficulty answering open‐ended questions should be explored. Examining the effectiveness of low‐technology communication aids, such as picture cards or line drawings, could facilitate the use of open‐ended questions, enabling individuals with limited verbal abilities to provide accurate and unbiased information (Pereira and Aldridge 2023; Hollomotz 2018). Further, allowing extended response times could improve responsiveness (Blackbourn and Baum 1987; Friedman and Spassiani 2024).

Further research is needed to replicate the present findings in real‐world investigative interviews. Specifically, since individuals with intellectual disability tend to have short attention spans (Chamberlain 1985), an excessively long narrative practice phase may adversely affect their concentration during the substantive interview phase. Therefore, future studies should aim to determine the optimal duration for which such individuals should be interviewed. The effectiveness of open‐ended prompts and narrative practices in eliciting information from those with severe disabilities is also likely to be limited, so devising strategies to conduct effective investigative interviews with victims with severe intellectual disability has academic and practical significance.

This study examined whether practising answering open‐ended questions during the early phases of an interview enhances the accuracy and quantity of information provided by individuals with intellectual disabilities. The findings suggest that narrative practices increases the amount of accurate details without raising inaccurate details, reinforcing the benefits of using open‐ended prompts in investigative interviews. However, it remains unclear whether narrative practices directly improves the accuracy of statements, as the increase in accurate details may be attributed to the overall quantity of information provided rather than an actual enhancement in accuracy. Additionally, limitations such as the severity of intellectual disability, individual differences in cognitive and linguistic abilities, and the study's sample characteristics should be considered when interpreting the results. Given these factors, further research is needed to explore the long‐term effectiveness of narrative practice in real‐world settings, determine the optimal duration of training, and assess its applicability for individuals with severe intellectual disabilities. Addressing these areas will help refine best practises for conducting effective and reliable investigative interviews with this population.

## Ethics Statement

This study was approved by the Institutional Review Board of Dongyang University (1041495‐201908‐HR‐01‐01). The legal guardians of the participants provided written informed consent prior to their inclusion in the study.

## Conflicts of Interest

The author declares no conflicts of interest.

## Data Availability

The dataset generated and analysed during the current study are available in the Zenodo repository, https://doi.org/10.5281/zenodo.11440278. This is not publicity available due to privacy concern, but are available from the corresponding author on reasonable request.

## Appendix A Questions Asked During the Narrative Practice and Substantive Interview Phases

**Table:**

Narrative practise phase *Condition 1 (practise with open‐ended questions)* 1. I'd like to know you better. What did you do last weekend? 2. Tell me everything that happened over the weekend, from the time you woke up until you went to bed. 3. [action] You mentioned you [an activity mentioned by the participant]. Tell me more about that. 4. [action] Tell me everything that happened after [the activity mentioned by the participant]. 5. [person] Earlier, you mentioned [a person mentioned by the participant]. Tell me more about [the person]. 6. [person] Tell me everything about [the person's] appearance. 7. [location] You mentioned [a location]. Tell me more about that. 8. [location] Tell me everything you know about [the location]. 9. [clothes] Describe the outfit you were wearing during the weekend. 10. [clothes] Tell me more about your [clothes]. *Condition 2 (practise with specific questions)* 1. I'd like to know you better. What did you do last weekend? 2. What was the first thing you did when you woke up? 3. [action] Of all the things you did last weekend, which activity was your favourite? 4. [action] Of all the things you did last weekend, which activity was your least favourite? 5. [person] Who was with you? 6. [person] Who was [the person mentioned by the participant]? 7. [location] Where were you? 8. [location] What do you like about [the location]? 9. [clothes] What were you wearing? 10. [clothes] What was the colour of your [pants/shirt]? Substantive interview phase *Open‐ended questions* 1. I understand that, a few hours ago, you met a photographer and did lots of things with the photographer. I wasn't there at the time. I'd like to know what happened. 2. Tell me what happened from the time you entered the room until you left the room. 3. You said you had a photo taken. Tell me more about when you had the photo taken. 4. Tell me everything the photographer did for you. 5. Please tell me everything about the photographer. 6. Tell me about the place where you had the photo taken. 7. Tell me everything about the costume you wore. *Specific and misleading questions (in turns)* 1. [specific] Who helped you wear the costume?” 2. [misleading] What was the colour of the dress worn by the photographer? 3. [specific] What did the photographer say to you? 4. [misleading] How did the photographer hug you? 5. [specific] What was depicted in the poster hung on the wall? 6. [misleading] What did the house in the poster look like? 7. [specific] What was the colour of the hat? 8. [misleading] What was the colour of the photographer's hat? 9. [specific] What was the colour of the photographer's outfit? 10. [misleading] Who helped you remove your socks? 11. [specific] How many photos did the photographer take? 12. [misleading] When did the photographer yell at you? 13. [specific] How did you feel? 14. [misleading] Why did you feel bad?

*Note:* The underlined words were misinformation.
